# Decoding the obesity–cancer connection: lessons from preclinical models of pancreatic adenocarcinoma

**DOI:** 10.26508/lsa.202302228

**Published:** 2023-08-30

**Authors:** Christian F Ruiz, Cathy Garcia, Jeremy B Jacox, Lauren Lawres, Mandar D Muzumdar

**Affiliations:** 1 Department of Genetics, Yale University School of Medicine, New Haven, CT, USA; 2 Yale Cancer Biology Institute, Yale University , West Haven, CT, USA; 3 Department of Medicine (Section of Medical Oncology), Yale University School of Medicine, New Haven, CT, USA; 4 Department of Immunobiology, Yale University School of Medicine, New Haven, CT, USA; 5 Yale Cancer Center, Yale University , New Haven, CT, USA

## Abstract

Obesity is a metabolic state of energy excess and a risk factor for over a dozen cancer types. Because of the rising worldwide prevalence of obesity, decoding the mechanisms by which obesity promotes tumor initiation and early progression is a societal imperative and could broadly impact human health. Here, we review results from preclinical models that link obesity to cancer, using pancreatic adenocarcinoma as a paradigmatic example. We discuss how obesity drives cancer development by reprogramming the pretumor or tumor cell and its micro- and macro-environments. Specifically, we describe evidence for (1) altered cellular metabolism, (2) hormone dysregulation, (3) inflammation, and (4) microbial dysbiosis in obesity-driven pancreatic tumorigenesis, denoting variables that confound interpretation of these studies, and highlight remaining gaps in knowledge. Recent advances in preclinical modeling and emerging unbiased analytic approaches will aid in further unraveling the complex link between obesity and cancer, informing novel strategies for prevention, interception, and therapy in pancreatic adenocarcinoma and other obesity-associated cancers.

## Abstract (precis)

Preclinical models demonstrate that obesity reprograms the pretumor or tumor cell and its micro- and macro-environments to drive cancer development via altered cellular metabolism, hormone dysregulation, inflammation, and microbial dysbiosis.

## Introduction

**Figure 1.**
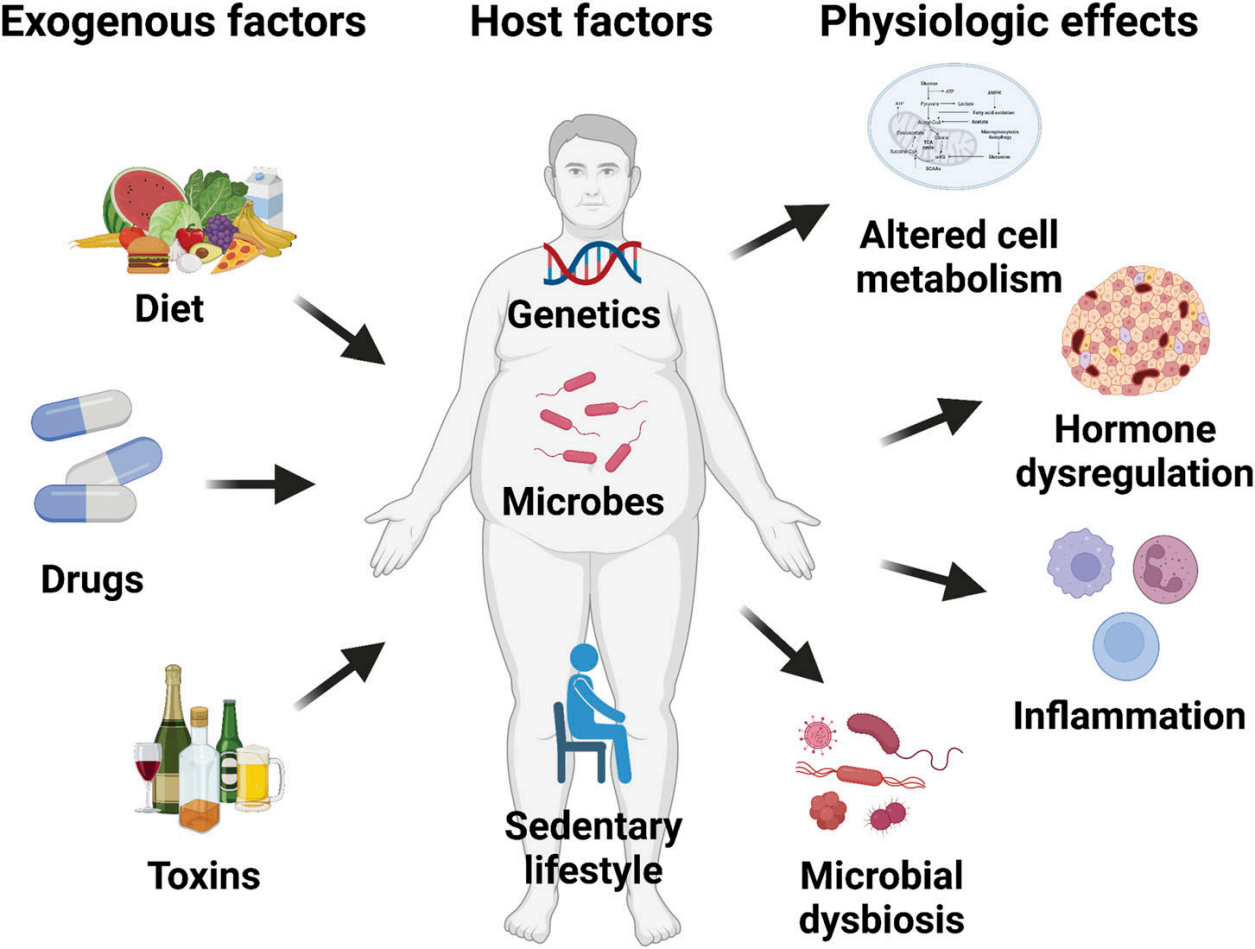
Causes and consequences of obesity. Graphical illustration of host and exogenous factors contributing to obesity and the resultant physiologic effects that promote cancer development.

**Figure 2.**
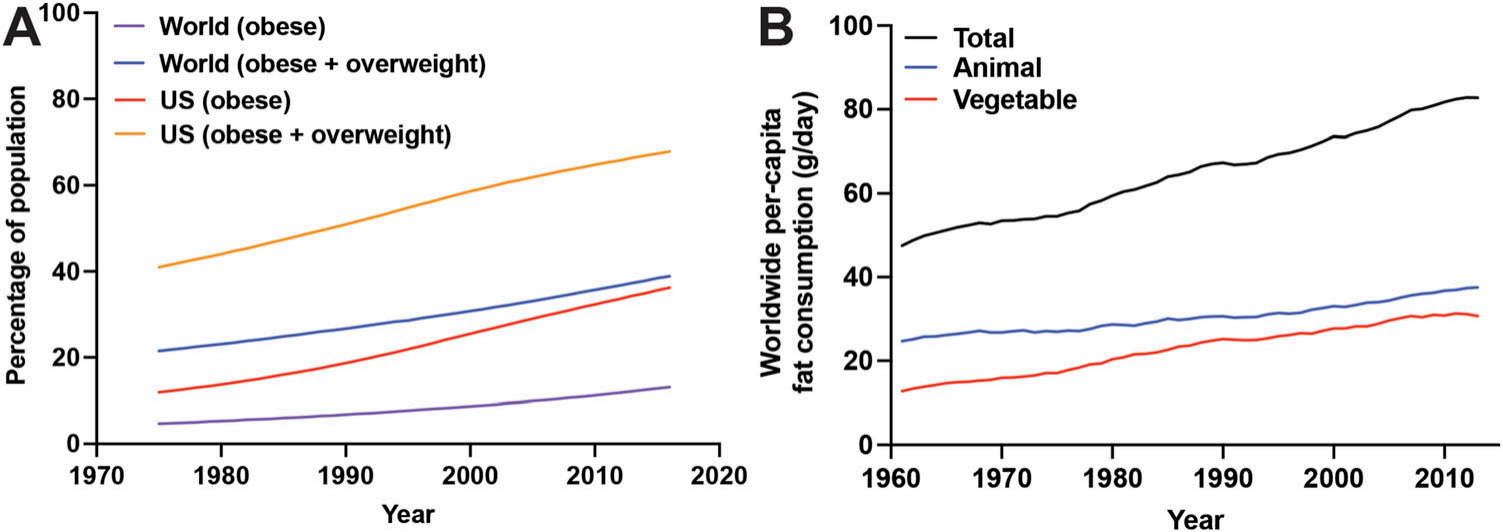
Worldwide trends in obesity rates and fat consumption. **(A)** The proportions of obese (body mass index>30) or obese + overweight (body mass index>25) individuals have increased rapidly worldwide and at a much more alarming rate in Western countries (e.g., United States (US)) over the last 50 yr. Data sourced from the World Health Organization. **(B)** Per-capita fat consumption (grams per day) by source (animal versus vegetable) over the last 60 yr. Data sourced from the Food Agricultural Organization of the World Health Organization.

Obesity is a metabolic state of energy excess associated with systemic alterations in host physiology including hormone dysregulation, insulin resistance, inflammation, and microbial dysbiosis. Despite years of research, there remains a lack of consensus on the causes of energy imbalance in obesity, which include both host (e.g., genetics, gut microbes, sedentary lifestyle) and exogenous factors (e.g., diet, drugs, toxins) (Fig 1). In contrast, there is little doubt that obesity plays a consequential role in numerous chronic diseases, spurring the World Health Organization and other major governmental health organizations to propose frameworks for obesity prevention. Although the impact of obesity on diabetes and cardiovascular disease is well established, it has become increasing clear that obesity is also a pathogenic driver of cancer. Indeed, obesity is associated with an increased risk of over a dozen cancer types, including nearly all cancers of the gastrointestinal tract (Lauby-Secretan et al, 2016), yet the mechanistic basis for these relationships remains incompletely understood. Given the rapid rise in the worldwide prevalence of obesity (Fig 2A), deciphering the mechanisms by which obesity promotes cancer development has become an urgent societal imperative that is likely to broadly impact human health.

In this review, we enumerate these mechanisms by focusing on evidence garnered from preclinical mouse models of cancer. We center this discussion on pancreatic adenocarcinoma (PDAC), the predominant histologic subtype of pancreatic cancer and a leading cause of cancer-related death worldwide. We chose PDAC as a paradigmatic example of an obesity-driven cancer for several compelling reasons. First, epidemiologic studies over the last two decades have consistently shown a positive dose-dependent association between Body Mass Index (BMI), a measure of obesity, and PDAC risk (Michaud et al, 2001; Larsson et al, 2007; Arslan et al, 2010; Klein, 2021). In fact, the continual rise in PDAC incidence is thought to be due, in large part, to a parallel increase in obesity rates, especially in younger individuals (Sung et al, 2019). Furthermore, higher pre-diagnostic BMI has been associated with increased metastasis at diagnosis and decreased PDAC survival across all clinical stages (Calle et al, 2003; Li et al, 2009; Yuan et al, 2013; Kasenda et al, 2014), arguing that obesity may also drive progression to more advanced disease. Second, unlike other more prevalent cancers, PDAC has not enjoyed the benefits of the targeted therapy and immunotherapy revolutions. Combination chemotherapy is the standard of care for nearly all patients with advanced disease, and long-term prognosis remains poor (Conroy et al, 2011; Von Hoff et al, 2013). Thus, a greater focus on identifying mechanisms that promote PDAC risk is necessary to understand how to prevent or intercept the disease. Third, PDAC is driven by a single hallmark oncogene, *KRAS*, in >90% of cases (Bailey et al, 2016; Cancer Genome Atlas Research Network, 2017), reducing the genetic variability required in preclinical models and potentially enhancing their generalizability. Indeed, Cre/LoxP-mediated conditional activation of oncogenic *Kras* expression in the mouse pancreas (Table 1) stimulates the formation of preinvasive pancreatic intraepithelial neoplasia (PanINs) capable of progressing to advanced PDAC (Aguirre et al, 2003; Hingorani et al, 2003; Hingorani et al, 2005; Guerra et al, 2007; Habbe et al, 2008; Kopp et al, 2012). These autochthonous genetically engineered mouse models (GEMMs) recapitulate the genetic and histologic features of the human disease, allowing the faithful study of the contributions of obesity to PDAC development. Finally, the pancreas plays a central role in response to feeding by secreting digestive enzymes (from exocrine acinar cells) and hormones (from endocrine islet cells) to aid in the absorption and cellular uptake of essential nutrients and to regulate glucose homeostasis. Therefore, the pancreas is uniquely positioned to be the great integrator of the effects of obesity. In the ensuing sections, we discuss relevant methods to model obesity in mice before embarking on a review of the evidence delineating putative mechanisms linking obesity to PDAC.

**Table 1.** Genetically engineered mouse models used in obesity studies of PDAC.

GEMM strains	Abbreviation	Pancreatic cell types induced	Timing of oncogene induction
*Pdx1-Cre;Kras* ^*LSL-G12D*^	*KC*	All	Embryonic (E8.5)
*Pdx1-Cre;Kras* ^*LSL-G12D*^ *; Trp53* ^*+/LSL-R172H*^	*KPC*	All	Embryonic (E8.5)
*Pdx1-Cre;Kras* ^*LSL-G12D*^ *;Lep* ^*ob/ob*^	*KCO*	All	Embryonic (E8.5)
*Ptf1a* ^*Cre*^ *;Kras* ^*LSL-G12D*^	*PK*	All	Embryonic (E9.5)
*Ptf1a* ^*CreERT*^ *;Kras* ^*LSL-G12D*^	*PK-ER*	Acinar cells	Tamoxifen; postnatal
*Elastase-CreERT2;Kras* ^*LSL-G12D*^	*EK-ER*	Acinar cells	Tamoxifen; postnatal
*Elastase-tTA; TetO-Cre;Kras* ^*LSL-G12V*^	*EK-Tet*	Acinar cells	Doxycycline (Tet-OFF); embryonic or postnatal
*Ck19* ^*CreERT*^ *;Dnmt1* ^*LSL-HrasG12V-IRES-GFP*^	*CH*	Duct cells	Tamoxifen; postnatal

LSL means LoxP-STOP-LoxP.

### Inducing obesity in cancer models

#### Genetic models of obesity

Both genetic and dietary models of obesity have been used to study PDAC pathogenesis and cancer more broadly. Genetic models have primarily centered on perturbation of the leptin signaling pathway, in which either the appetite-suppression hormone leptin (*Lep*^*ob/ob*^) or its receptor (*Lepr*^*db/db*^) is deficient, leading to rapid onset obesity from overfeeding (Friedman, 2019). Both *Lep*^*ob/ob*^ and *Lepr*^*db/db*^ accelerated oncogenic *Kras*-driven tumor progression in *Pdx1-Cre;Kras*^*LoxP-STOP-LoxP-G12D*^ (*KC*) mice and enhanced tumor growth and metastasis in syngeneic transplant models (Zyromski et al, 2009; Incio et al, 2016a; Chung et al, 2020). Although monogenic forms of obesity—including mutations in the *LEP* and *LEPR* genes—are rare in humans, genome-wide association studies of polygenic (common) obesity largely revealed genes in the leptin–melanocortin 4 receptor (MC4R) hypothalamic appetite control axis or other nodes in central control of body weight by the brain (Loos & Yeo, 2022). Therefore, *Lep*^*ob/ob*^ and *Lepr*^*db/db*^ mice may be viewed as reasonable models to study obesity-driven cancer. In contrast, these mice suffer from corollary effects of reduced energy expenditure because of starvation physiology, including infertility, immune dysfunction, and neurohormonal dysregulation (e.g., hypercortisolemia), which may confound the interpretation of tumor phenotypes (Friedman, 2019). Nonetheless, leptin signaling mutants offer three distinct advantages compared with the dietary approaches described below: (1) a controlled diet, as obesity is because of overeating standard chow without varying macronutrient composition (e.g., fat); (2) faster and more extreme weight gain; and (3) reversibility. Our group induced early rapid weight loss in *KC;Lep*^*ob/ob*^ (*KCO*) mice either by restoring leptin (using an adeno-associated viral vector) or reducing dietary intake (caloric restriction). As both interventions intercepted tumor development (Chung et al, 2020), neither leptin deficiency itself nor the starvation physiology that results drive the enhanced tumor phenotype. In contrast, weight loss at a later timepoint—when most mice have already developed advanced tumors—did not prolong survival (Chung et al, 2020). These experiments demonstrate that the pro-tumorigenic effects of obesity can be reversed in a stage-specific manner, advocating for interventions to prevent or intercept pancreatic cancer early during tumorigenesis.

#### Diet-induced models of obesity

Diet-induced obesity studies using high-fat diets (HFD) predominate in preclinical research of obesity-driven cancer. In support of this approach, human epidemiologic studies have demonstrated a strong association between excess dietary fat and increased PDAC risk (Soler et al, 1998; Thiébaut et al, 2009; Zhang et al, 2009; Taunk et al, 2016; Gordon-Dseagu et al, 2017; Zheng et al, 2017). Several independent groups have shown that HFD feeding increases the formation of PanINs, progression to PDAC, and metastasis in oncogenic *Kras*-driven autochthonous mouse models and syngeneic transplant models (Khasawneh et al, 2009; Dawson et al, 2013; Philip et al, 2013; Incio et al, 2016a; Incio et al, 2016b; Chang et al, 2017; Zaytouni et al, 2017; Zhang et al, 2019; Sun et al, 2022), consistent with a causal role for excess fat consumption in pancreatic tumorigenesis. Similarly, HFD feeding enhanced carcinogen-induced pancreatic tumor formation in rats and hamsters, indicating that the tumor-promoting effects of excess fat are sustained across species and genetic drivers (Birt et al, 1989; Z’Graggen et al, 2001; Hori et al, 2011).

However, HFDs from study to study were not identical, varying in fat source, fatty acid composition, caloric content, and nonfat constituents (e.g., sugars and protein) (Table 2). Currently, there are many commercially available HFDs that make rodents obese, with 45% and 60% kcal lard-based diets, originally formulated by Research Diets, Inc., making up the lion’s share of HFDs used in research studies (Speakman, 2019). Mice become obese on a 45% kcal HFD; however, the 60% kcal HFD is far more frequently used (Table 2) because of faster onset obesity. This fat proportion may be considered excessive compared with the normal range (10–18% kcal fat) in chow given to laboratory mice and far exceeds even the upper quartile of human fat consumption of 45% (Guasch-Ferre et al, 2017). Importantly, an increased percentage of calories from fat is associated with a concomitant reduction in sugar content, which could confound results, as human epidemiologic studies have linked excess sugar intake to PDAC risk (Larsson et al, 2006). A recent study comparing a high-carbohydrate diet (72% versus 62% kcal with excess sucrose and dextrin) showed more modest effects on weight gain and tumor development compared with a lard-based HFD (60% kcal fat) in *Elastase-CreERT2*;*Kras*^*LoxP-STOP-LoxP-G12D*^ (*EK-ER*) (Zhu et al, 2022). These results indicate that excess fat is not only more obesogenic (Hu et al, 2018) but also more tumor-promoting in mice. Excess intake of specific sugars (e.g., sucrose or fructose) enhanced tumor formation in mouse models of other cancer types (e.g., colorectal cancer and acinar cell carcinoma; [Goncalves et al, 2019; Dooley et al, 2020]). It remains to be seen whether particular sugars also drive PDAC development.

**Table 2.** High-fat diet formulations used in obesity studies of PDAC.

Preclinical pancreatic cancer study	Commercial vendor	Product numbers	% fat	Fat sources
Khasawneh et al (2009)	Research Diets, Inc.	D12492	60	Lard
Dawson et al (2013)	Dyets, Inc.	NR	40	Corn oil
Philip et al (2013)	Lab Supply	58Y1	60	Lard
Incio et al (2016a)	Research Diets, Inc.	D12492	60	Lard
Incio et al (2016b)	Research Diets, Inc.	D12492	60	Lard
Chang et al (2017)	Dyets, Inc.	NR	40	Corn oil
Gomez-Chou et al (2017)	Lab Supply	58Y1	60	Lard
Zaytouni et al (2017)	Research Diets, Inc.	D12492	60	Lard
Chang et al (2018)	Dyets, Inc.	NR	40	Corn oil
Dong et al (2019)	Dyets, Inc.	NR	40	Corn oil
Zhang et al (2019)	Research Diets, Inc.	D12492	60	Lard
Lupo et al (2020)	NR	NR	60	NR
Lien et al (2021)	Envigo	TD.200397, TD.200396	57-90	Lard, palm oil, soybean oil
Takenaga et al (2021)	Research Diets, Inc.	D12492	60	Lard
Fonteneau et al (2022)	Research Diets, Inc.	D12492	60	Lard
Sun et al (2022)	NR	NR	60	NR
Zhang et al (2022)	Research Diets, Inc.	D12492	60	Lard
Zhu et al (2022)	Test Diet	58Y1	60	Lard
Pita-Grisanti et al (2023) *Preprint*	Test Diet	58Y1	60	Lard
Wang et al (2023)	Research Diets, Inc.	D12492	60	Lard
Zhang et al (2023) *Preprint*	Research Diets, Inc.	D12492	60	Lard

NR means Not Reported.

Similar to sugars, all fat sources are not necessarily identical in promoting tumorigenesis, as specific fatty acids are known to exert unique effects on tumor cells and their environment (Hardy et al, 2000; Holzer et al, 2011; Kamphorst et al, 2013; Magtanong et al, 2019). Human epidemiologic studies have limited power to delineate specific fatty acids as cancer drivers because of recall bias, lack of granularity in diet surveys, or small sample sizes. Importantly, traditional lard-based HFDs used in mouse cancer studies (Table 2) do not represent the increasing human consumption of vegetable oils worldwide (Fig 2B). Therefore, systematic studies comparing the effects of different dietary fat sources on PDAC development in preclinical models are sorely needed. Recent efforts have identified the differential roles of saturated, monounsaturated, and polyunsaturated fats in advanced tumor growth and metastasis in models of various cancers (Ubellacker et al, 2020; Lien et al, 2021; Pascual et al, 2021; Altea-Manzano et al, 2023), though the contributions of specific dietary fatty acids in tumor initiation and early progression remain unknown. Encouragingly, Research Diets, Inc., has developed a panel of over 30 diets ranging across the spectrum of human macronutrient consumption (Hu et al, 2018), which may help address this knowledge gap.

#### Additional considerations for modeling obesity in cancer studies

Several other confounding variables must be carefully considered and reported when studying obesity-driven cancer in mice. First, HFDs exert strain-specific effects on systemic metabolism. For example, lard-based HFDs readily induced weight gain, insulin resistance, hepatic steatosis, and glucose intolerance in C57BL/6 and 129X1/SvJ mice, whereas BALB/c mice were more resistant to these metabolic phenotypes (Birt et al, 1989; Andrikopoulos et al, 2005; Berglund et al, 2008; Montgomery et al, 2013; Smith et al, 2019). Second, mouse sex influences the effects of particular HFDs, as female mice are more resistant to weight gain and PDAC development when fed a lard-based HFD (Chang et al, 2017). In contrast, no significant differences in survival or tumorigenesis were observed comparing genetically obese (*Lep*^*ob/ob*^) male and female *KCO* mice fed standard chow (Chung et al, 2020). Third, the amount of food consumed may vary considerably between individual mice, confounding results through differential weight gain and variable effective doses of specific dietary components (e.g., fatty acids or sugars). Similarly, interventions that block weight gain (e.g., metformin [Chang et al, 2018]) may simply subvert tumorigenesis by preventing obesity rather than addressing a specific mechanism of obesity-driven cancer (e.g., hyperinsulinemia). Finally, emerging human data have shown that physical activity and exercise reduce PDAC risk (Farris et al, 2015), and an aerobic exercise intervention decreased PDAC tumor growth in mice (Kurz et al, 2022). Obese mice exhibit lower physical activity, and it remains unclear how much of the tumor-promoting phenotype of obesity could be attributed to a more sedentary nature. Therefore, whether and how increased physical activity impedes tumorigenesis in obese mice remains to be elucidated, though newer studies are exploring these relationships (Pita-Grisanti et al, 2023 *Preprint*). Despite the plethora of intrinsic (e.g., sex, genetic mutations, strain) and environmental (e.g., HFD amount and composition, physical activity intervention) variables that could confound results, the totality of preclinical studies to date support the conclusion that obesity—mediated both by how much and what is eaten—is a causal risk factor in PDAC development and provide critical in vivo models to uncover mechanisms of obesity-driven cancer.

### Investigating mechanisms of obesity-driven cancer

#### Obesity as a permissive factor for tumorigenesis

For cancers to arise, cells must acquire the capacity for sustained proliferation while overcoming both intrinsic evolutionary constraints and constraints imposed by the host environment. These properties may be attained through the sequential acquisition of genetic mutations in proto-oncogenes (e.g., *KRAS*) and tumor-suppressor genes (e.g., *TP53*) that promote cancer initiation, progression, and maintenance (Hanahan & Weinberg, 2011; Vogelstein et al, 2013; Bailey et al, 2018). Yet, it has become increasingly clear that mutations alone are frequently incapable of driving tumorigenesis. For example, clonally expanded cell populations harboring cancer-associated mutations have been observed in numerous physiologically normal human tissues (Jaiswal et al, 2014; Martincorena et al, 2015; Yokoyama et al, 2019; Hill et al, 2023). The presence of these “driver” mutations only predisposes to malignancy (Genovese et al, 2015), but does not ensure it—arguing that they are permissive but insufficient for cancer development. It has therefore been postulated that non-mutational adaptations must cooperate with such mutations to induce cancer (Alizadeh et al, 2015).

One such factor is obesity, which has pleiotropic effects on tumor cells and the micro- and macro-environments in which they reside, all of which may promote the development of cancer. Although obesity-related oxidative stress may induce mutagenesis, published studies do not support this mechanism in PDAC; there is no significant correlation between obesity and the acquisition of additional cancer driver mutations in humans and GEMMs (Chang et al, 2017; Chung et al, 2020). We have further shown that *KCO* mice have comparable tumor latency and survival with mice harboring an additional driver *Trp53* mutation (e.g., *Pdx1-Cre;Kras*^*LoxP-STOP-LoxP-G12D*^*;Trp53*^*+/LoxP-STOP-LoxP–R172H*^ [*KPC*]) (Muzumdar et al, 2016; Chung et al, 2020), arguing that obesity can effectively substitute for a second genetic hit in tumor progression. Given the strong association between obesity and cancer risk, we hypothesize that obesity reprograms pretumor cells (cells-of-origin) either directly or via reciprocal interactions with their micro- and macro-environments. This leads to changes in the pretumor cell epigenome, metabolome, signaling or immune cell/microbe interactions, which make them competent for transformation in the right genetic context (e.g., *KRAS* mutation for PDAC).

In the ensuing sections, we review putative mechanisms by which obesity primes the pancreas for tumorigenesis via (1) altered cellular metabolism, (2) hormone dysregulation, (3) inflammation, and (4) microbial dysbiosis (Fig 1). Although we touch on data from experiments involving transplantation (subcutaneous or orthotopic) of advanced PDAC cells into obese preclinical models, the strongest human epidemiologic data to date support a pathogenic role for obesity in PDAC risk (tumor initiation and early progression), which is not adequately modeled by these approaches. Newer organoid culture methods that allow the isolation, propagation, and transplantation of normal ducts or PanINs into HFD-fed or genetically obese mice offer a more relevant and tractable approach for studying early events in tumorigenesis (Boj et al, 2015; Lupo et al, 2020), though they have only recently been adopted more broadly in cancer research (Tuveson & Clevers, 2019). Therefore, we emphasize supportive evidence from autochthonous GEMMs of PDAC, which enable analyses of how obesity shapes tumor cells and their microenvironment from precancerous stages all the way through to advanced malignancy.

#### Altered cellular metabolism

**Figure 3.**
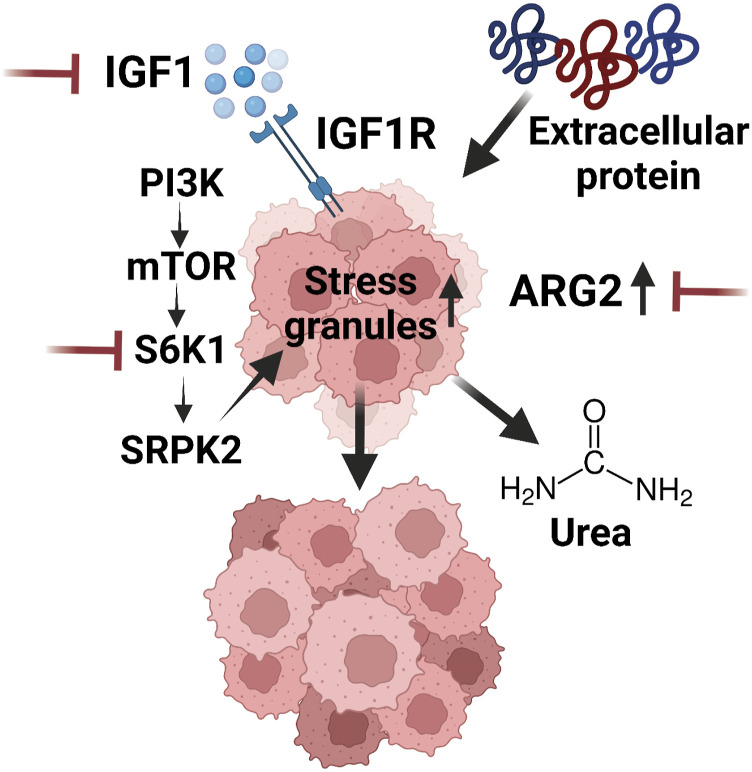
Obesity-associated metabolic alterations in advanced PDAC tumors. PDAC tumors grown in HFD-fed mice exhibit increased tumor growth and dependency on nitrogen flux through arginase (ARG2) and stress granule formation via a signaling axis encompassing insulin-like growth factor 1 (IGF1), phosphoinositide 3-kinase (PI3K), mammalian target of rapamycin (mTOR), S6 kinase (S6K1), and serine–arginine protein kinase 2 (SRPK2). Nodes for inhibition that suppress tumor growth in vivo are noted in red.

Tumor cells must undergo extensive metabolic reprogramming to acquire the energy and building blocks required for proliferation and survival (Encarnación-Rosado & Kimmelman, 2021; Halbrook et al, 2023). Obesity can perturb cellular metabolism by modulating the availability of metabolites and energy sources in the host environment. Much of the work identifying metabolic pathways associated with obesity in PDAC has used transplantation of advanced PDAC cells into HFD-fed mice. For example, HFD induced the expression of arginase (ARG2) in orthotopic PDAC transplant tumors, concordant with higher ARG2 protein levels in tumor biospecimens from high-BMI patients (Zaytouni et al, 2017). ARG2 lies in a metabolic pathway that converts arginine into ornithine and urea, an important amino acid catabolic pathway. PDAC cells efficiently scavenge extracellular proteins in vitro and in vivo as a means to support their growth (Commisso et al, 2013; Kamphorst et al, 2015; Davidson et al, 2017), requiring efficient shuttling of nitrogenous waste through the urea cycle. Indeed, metabolic tracing of labelled amino acids revealed greater nitrogen flux into the urea cycle in tumors grown in obese mice. Consequently, ARG2 inhibition led to ammonia accumulation and suppressed tumor growth (Zaytouni et al, 2017). A more recent study demonstrated that obesity also induces stress granule formation via IGF1/PI3K/S6K1 signaling in orthotopic PDAC transplant models and that inhibition of stress granule formation at multiple nodes along this pathway impedes obesity-driven tumor growth in vivo (Fonteneau et al, 2022). These studies implicated multiple unique metabolic dependencies acquired by advanced PDAC tumors growing in the context of obesity (Fig 3). However, as most patients with PDAC experience weight loss (cachexia), anorexia, and reduced appetite, the translational relevance of preclinical studies feeding a HFD to mice transplanted with advanced tumor cell lines is unclear.

Emerging data have revealed several metabolic determinants of PDAC initiation, including changes in glucose metabolism, amino acid utilization, autophagy, mitochondrial reactive oxygen species production, and lipid metabolism, many of which can be independently modulated by HFD feeding (Khasawneh et al, 2009; DeNicola et al, 2011; Carrer et al, 2017; Vernucci et al, 2019). Autophagy—a metabolic process that removes damaged cellular components—was shown to suppress tumor formation in *KC* mice, as knockout of essential autophagy genes (*Atg5* or *Atg7*) increased the number of low-grade PanINs (Rosenfeldt et al, 2013; Yang et al, 2014; Long et al, 2022). In contrast, autophagy inhibition interfered with progression to and maintenance of advanced PDAC (Yang et al, 2011; Rosenfeldt et al, 2013; Yang et al, 2014; Yang et al, 2018), supporting a stage-specific role for autophagy in pancreatic tumorigenesis. A similar context-dependent function for cholesterol metabolism in PDAC development has been described. Conditional knockout of the rate-limiting enzyme in distal cholesterol biosynthesis (Nsdhl) diminished oncogenic *Kras*-driven tumor progression in mice, but PDACs that emerged exhibited a basal differentiation phenotype harboring an epithelial-to-mesenchymal (EMT) transcriptional program induced by an SREBP1/TGFβ autocrine signaling axis (Gabitova-Cornell et al, 2020). Consistent with these data, inhibition of sterol synthesis using HMG-CoA reductase inhibitors (statins) impeded oncogenic *Kras*-induced acinar-to-ductal metaplasia (ADM), an early prerequisite step in pancreatic tumorigenesis preceding PanIN formation (Kopp et al, 2012; Storz, 2017; Carrer et al, 2019). Acetyl-CoA—a key input into the mevalonate pathway that results in sterol synthesis —was found to be a critical mediator of ADM, as knockout of acetyl-CoA lyase (*Acly*) reduced ADM and tumorigenesis in *KC* mice (Carrer et al, 2019). A separate study illuminated additional metabolic vulnerabilities associated with rewired cholesterol metabolism in advanced PDAC, as genetic deletion of *Soat1*—an enzyme involved in suppressing feedback responses due to hyperactive cholesterol biosynthesis—impaired the growth of orthotopically transplanted tumor organoids in vivo (Oni et al, 2020). Pre-malignant tissue has also been shown to impose reciprocal metabolic shifts on the host. Elevated levels of circulating branched-chain amino acids and adipose tissue wasting and cachexia have been observed as consequential early metabolic events in PDAC development, underscoring their potential as metabolic biomarkers in patients (Mayers et al, 2014; Danai et al, 2018). Collectively, these studies highlight the importance of pre-malignant metabolic transformations that support cancer development (Brunner & Finley, 2023). Convincing evidence directly linking obesity to PDAC pathogenesis via these metabolic pathways in preclinical models, however, is limited. Therefore, whether similar metabolic alterations occur in pretumor and tumor cells early in obesity-driven tumorigenesis remains to be defined.

#### Hormone dysregulation

Clinical and preclinical studies to date have implicated obesity-induced alterations in the levels of insulin, gastrointestinal peptide hormones, and adipocyte-derived hormones (adipokines) in PDAC development. Higher circulating levels of insulin and its precursor proinsulin (measured as C-peptide) have been associated with increased PDAC risk in human cohorts (Wolpin et al, 2013). Although insulin has long been thought to be a critical mediator of obesity-driven cancer, in vivo evidence for a role in early tumor development in faithful cancer models has only recently emerged (Wang et al, 2018; Zhang et al, 2019). In response to glucose, insulin is synthesized and secreted by endocrine β cells of the islets of Langerhans, which lie in close proximity to acinar cells of the exocrine pancreas, a putative cell-of-origin of PDAC (Kopp et al, 2012; Storz, 2017). Recent studies have demonstrated bidirectional blood supply between the endocrine and exocrine pancreas (Dybala et al, 2020) and acinar cell heterogeneity within the peri-islet region (Egozi et al, 2020; Tosti et al, 2021), substantiating a potential role for endocrine–exocrine hormonal signaling in health and disease (Mastracci et al, 2022). Obesity (HFD and *Lep*^*ob/ob*^-induced) enhances basal (fasting) levels of insulin in circulation in mice (Zhang et al, 2019; Chung et al, 2020). Furthermore, metformin, a mainstay first-line antidiabetic, prevented hyperinsulinemia and slowed tumor development including progression to advanced PanINs and PDAC in a faithful oncogenic *Kras*-driven autochthonous model (*PK*) fed a high-fat, high-calorie diet (Chang et al, 2018). However, metformin had a multitude of additional effects including abrogating weight gain, adiposity, hepatic steatosis, and oncogenic signaling in the pancreas, confounding the interpretation that normalizing insulin levels were the driving force behind its anti-tumor effects. Indeed, in a separate study, metformin suppressed PanIN formation in *KC* mice fed normal chow (Chen et al, 2017), suggesting that how metformin impedes tumorigenesis may not be dependent on obesity.

To directly test whether obesity-induced hyperinsulinemia enhances pancreatic tumorigenesis, the Johnson and Kopp groups lowered insulin gene dosage in *Ptf1a*^*CreERT*^;*Kras*^*LoxP-STOP-LoxP-G12D*^ (*PK-ER*) mice, allowing acinar cell-specific activation of oncogenic *Kras*. Mice have two insulin genes (*Ins1* and *Ins2*) that both contribute to insulin production in the pancreas, thus complicated genetic crosses were required to compare mice retaining one versus two copies of a single insulin gene. Strikingly, *PK-ER;Ins2*^*−/−*^*;Ins1*^*+/−*^ mice (retaining a single copy of *Ins1*) exhibited reduced insulin levels and PanIN formation when fed a HFD compared with *PK-ER;Ins2*^*−/−*^*;Ins1*^*+/+*^ mice (Zhang et al, 2019), arguing that HFD promotes pancreatic tumorigenesis via induction of hyperinsulinemia. It should be noted that *Ins1* heterozygous mice did not gain as much weight on a HFD as their *Ins1* WT counterparts, confounding this interpretation. Furthermore, the converse experiment—in which differential gene dosage of *Ins2* was tested (*PK-ER;In1*^*−/−*^*;Ins2*^*+/−*^ versus *PK-ER;Ins1*^*−/−*^*;Ins2*^*+/+*^)—showed very little overall tumor burden without a significant difference between genotypes (Zhang et al, 2022). More convincing evidence has emerged from the same groups using conditional inactivation of the insulin receptor (InsR). *PK-ER;InsR*^*flox/flox*^ mice exhibited adequate weight gain and hyperinsulinemia but reduced PanIN formation in response to HFD feeding (Zhang et al, 2023 *Preprint*). Mechanistically, insulin induced metaplastic ductal conversion of primary acinar cells (ADM) in an InsR-dependent manner. Together, these data lend support to the insulin hypothesis that obesity-induced hyperinsulinemia drives the development of PDAC.

Our work has demonstrated that insulin is not the only β cell hormone capable of promoting PDAC development. We and others have found that prolonged obesity in *Lep*^*ob/ob*^ mice stimulated increasing β cell expression of the peptide hormone cholecystokinin (CCK) (Lavine et al, 2010; Chung et al, 2020). We further showed that islet CCK expression was concordant with reduced insulin expression, β cell dysfunction, and compromised glucose-stimulated insulin secretion (Chung et al, 2020). CCK is normally expressed by duodenal enteroendocrine cells, traverses the enterohepatic circulation, and acts on pancreatic acinar cells to induce digestive enzyme secretion. The Smith group showed that treatment with a competitive CCK receptor (CCKAR) antagonist suppressed tumor initiation in non-obese *KC* mice and that HFD feeding could promote tumor growth in a CCKAR-dependent manner in an orthotopic transplant model (Smith et al, 2014; Nadella et al, 2018). Moreover, chronic administration of the CCK analogue cerulein was sufficient to induce chronic pancreatitis—a known risk factor for PDAC—and accelerate tumorigenesis in *Elastase-tTA;TetO-Cre;Kras*^*LoxP-STOP-LoxP-G12V*^ (*EK-Tet*) mice (Guerra et al, 2007). More recent studies have shown that acute high doses of cerulein promote inflammation and epigenetic reprogramming of pancreatic acinar cells enhancing transformation by oncogenic *Kras* in lean mice (Alonso-Curbelo et al, 2021; Del Poggetto et al, 2021). Therefore, we hypothesized that physiologic β cell-derived CCK could be a local driver of tumorigenesis in obesity via endocrine (islet)–exocrine (acinar cell) signaling. Indeed, we confirmed that CCK was endogenously expressed in β cells of obese tumor-bearing *KCO* mice and that transgenic β cell CCK expression was sufficient to promote *Kras*-driven ductal tumorigenesis (Chung et al, 2020). Importantly, β cell CCK expression did not induce overt inflammation; instead, it promoted acinar cell proliferation in vivo (Chung et al, 2020) and ADM in vitro (Ardito et al, 2012). Furthermore, glucose reduction (via inhibition of the sodium-glucose like transporter 2) improved β cell function and suppressed islet CCK expression and tumor development in the *KCO* model (Chung et al, 2020), arguing that interventions that attenuate obesity-induced β cell stress may subvert tumorigenesis.

Taken together, these data suggest that multiple β cell hormones play a role in PDAC pathogenesis likely by directly or indirectly stimulating cell state transitions (e.g., ADM) permissive for tumorigenesis. Whether additional obesity-induced β cell hormones are also pathogenic remains to be seen. Given their endocrine function, β cells can signal to all body tissues; thus, β cell hormone dysregulation may impact tumor formation far beyond the pancreas. In contrast, the local concentrations of these hormones are likely to be highest within the pancreas itself, arguing for a preferential role in pancreatic tumorigenesis. The precise cellular and molecular mechanisms that lead to islet hormone dysregulation (including CCK expression) remain to be fully elucidated but could unlock novel opportunities for targeting the β cell to prevent or intercept exocrine tumorigenesis.

Beyond pancreatic β cells, another important endocrine cell type perturbed by obesity is the adipocyte, a fat-storage cell present in excess in obesity. Adipocytes secrete hormones (adipokines) that regulate important physiologic functions ranging from appetite to immunity. The adipokine leptin is secreted proportional to fat mass, as overweight and obese individuals exhibited elevated levels of circulating leptin compared with those of normal weight, whereas weight loss reduced leptin levels (Considine et al, 1996). Furthermore, circulating levels of leptin were positively correlated with PDAC risk in prospective human cohorts (Stolzenberg-Solomon et al, 2015; Babic et al, 2016). Despite evidence from in vitro studies supporting a mitogenic role for leptin in PDAC cells (Mendonsa et al, 2015; Harbuzariu et al, 2017), our data showed that obesity can accelerate pancreatic tumorigenesis in vivo in the absence of leptin (*Lep*^*ob/ob*^) or its receptor (*Lepr*^*db/db*^) (Chung et al, 2020), arguing against leptin itself being an essential driver of obesity-associated PDAC development.

Unlike leptin, preclinical models support a more significant role for the adipokine lipocalin-2 (Lcn2) in obesity-driven PDAC. Lcn2 has multiple functions including iron and lipid trafficking, appetite suppression (via binding of MC4R), and inflammation (via SLC22A17) (Gumpper et al, 2020). In human and mouse studies, adipocyte Lcn2 expression and/or circulating Lcn2 levels correlated with obesity, insulin resistance, inflammation, and early PDAC development (Wang et al, 2007; Yan et al, 2007; Moniaux et al, 2008; Catalán et al, 2009; Auguet et al, 2011). To evaluate the function of Lcn2 in pancreatic tumorigenesis, the Cruz-Monserrate group knocked out murine *Lcn2* (*Lcn2*^*−/−*^) in *EK-ER* mice and observed a significant reduction in HFD-driven tumor development and associated inflammation and fibrosis in the tumor microenvironment (Gomez-Chou et al, 2017). Importantly, *Lcn2* knockout decreased weight gain and had an impact on survival independent of HFD feeding, indicating that Lcn2 is required for tumorigenesis even in the absence of obesity. Concordant with this observation, orthotopic transplantation of advanced PDAC cells (*KPC*) into lean *Lcn2*^*−/−*^ mice delayed tumor growth and decreased inflammation and fibrosis. Mechanistically, Lcn2 stimulated pancreatic stellate cells (PSCs) to secrete proinflammatory cytokines in a Slc22a17-dependent manner (Gomez-Chou et al, 2017). Other studies in PDAC have demonstrated an important role for Lcn2 in cancer cachexia. Specifically, *Lcn2*^*−/−*^ mice were resistant to weight loss after implantation with syngeneic PDAC cells, whereas reintroduction of Lcn2 expression from bone marrow-derived cells was sufficient to induce MC4R-mediated appetite suppression in the hypothalamus and restore cachexia (Olson et al, 2021). Taken together, these data show that Lcn2 promotes PDAC progression and associated cachexia, though these effects are not clearly linked to obesity.

Apart from increasing the secretion of pro-tumorigenic adipokines, obesity may drive tumor development by lowering the expression of putative tumor-suppressive adipocyte hormones. One such hormone is adiponectin, which acts on its target receptors (AdipoR1/2) to lower glucose, improve insulin resistance, and induce fatty acid oxidation (Yamauchi et al, 2003). Circulating adiponectin levels were reduced in obesity, increased by weight loss in obese individuals, and inversely correlated with PDAC risk in humans (Hu et al, 1996; Grote et al, 2012; Bao et al, 2013b; De Rosa et al, 2013). Most studies exploring the effects of adiponectin, however, have used transplant models of advanced PDAC cells to demonstrate that activation of adiponectin signaling (e.g., with the agonist AdipoRon) decreases orthotopic tumor growth by opposing leptin action (Messaggio et al, 2017). Consequently, HFD feeding and associated hyperleptinemia antagonized the effects of AdipoRon on tumor growth and angiogenesis (Takenaga et al, 2021). Given the lack of adiponectin/AdipoR modulation in faithful autochthonous models of PDAC, a clear suppressive function for adiponectin in tumor initiation and early progression remains to be established.

**Figure 4.**
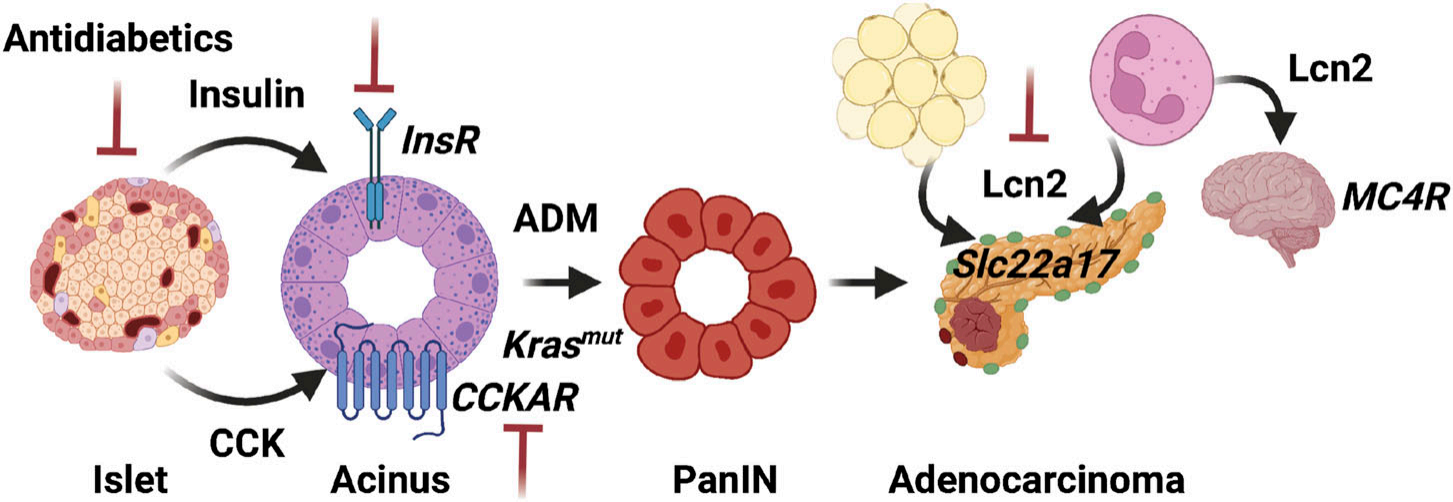
Obesity-associated hormone dysregulation as a driver of tumorigenesis. Aberrant expression of endocrine hormones promotes tumorigenesis at various stages of development. β cell hormones (insulin and CCK) act directly on acinar cell receptors (InsR and CCKAR, respectively) to promote acinar-to-ductal metaplasia (ADM), a prerequisite step in early PDAC development. Lcn2 from adipocytes and/or neutrophils (polymorphonuclear cells) induces tumor growth and cachexia via distinct receptors (Slc22a17 and MC4R, respectively). Potential nodes for inhibition that suppress early tumor development or advanced tumor growth in vivo are noted in red.

In summary, hormone dysregulation of both pancreatic β cells and adipocytes in obesity can drive PDAC development (Fig 4). Evidence from preclinical studies is most supportive of tumor-modulating roles for insulin, CCK, and Lcn2, whereas data are less conclusive for the adipokines leptin and adiponectin, despite correlations from human epidemiologic studies. Whether additional obesity-induced hormones from pancreatic islets, adipocytes, or other endocrine organs (e.g., pituitary or adrenal glands) drive pancreatic tumorigenesis remains to be elucidated. Although blocking hormone signaling as a prevention strategy has great appeal, execution is likely to be challenging. Targeting insulin signaling or its downstream effectors (e.g., phosphoinositide 3-kinase) is fraught with adverse metabolic effects and prone to feedback signaling reactivation, as has been shown in PDAC models (Hopkins et al, 2018). Targeting Lcn2 is similarly not straightforward because of the multitude of cellular sources (neutrophils, adipocytes, hepatocytes, tumor cells) and receptors (Slc22a17, MC4R, megalin) (Gumpper et al, 2020). On the other hand, CCK receptor antagonists have been tested in clinical studies without significant adverse effects, and *CCK* and *CCKAR* knockout mice are viable (Kopin et al, 1999; Lacourse et al, 1999; Dufresne et al, 2006). However, the requirement for β cell CCK in obesity-driven PDAC has not yet been firmly established in preclinical models and competitive CCK receptor antagonists may not be potent enough to counter the presumably high local concentrations of obesity-induced CCK within the pancreas (Chung et al, 2020). Alternatively, understanding and blocking the upstream mechanisms by which obesity alters the expression of these pro-tumorigenic hormones may offer another potential avenue to subvert their pathogenic roles in cancer.

#### Inflammation and modulation of anti-tumor immunity

**Figure 5.**
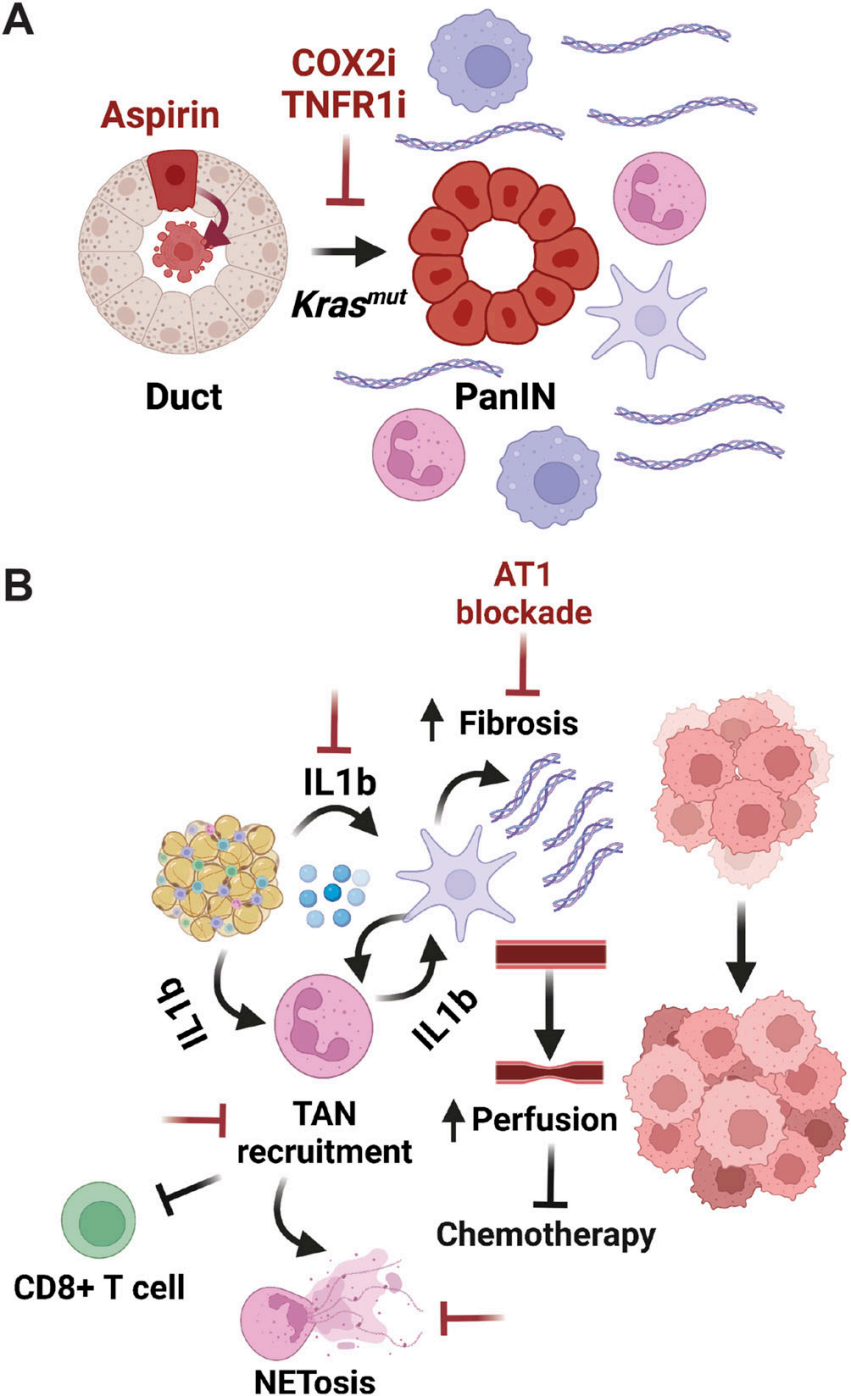
Obesity-induced inflammation as a driver of tumor initiation and growth of PDAC. **(A)** PanIN formation in obese mice is associated with increased myeloid infiltratration (denoted by pink neutrophils and blue macrophages) and fibrosis (denoted by purple stellate cell and collagen fibers). This can be blocked by targeting inflammatory pathways including COX2 and TNFα/TNFR1 signaling. Aspirin induces expulsion of Ras-transformed pancreatic duct cells (red). **(B)** Reciprocal IL1β-mediated interactions between adipocytes, tumor-associated neutrophils (TANs), and pancreatic stellate cells (purple) induce fibrosis, reduce perfusion impairing chemotherapeutic response, promote NETosis (formation of neutrophil extracellular traps), suppress cytotoxic CD8^+^ T cell infiltration, and in turn, promote tumor growth. Potential nodes for inhibition that suppress tumor growth in vivo are noted in red.

Obesity is frequently described as a state of chronic inflammation, as circulating inflammatory markers correlate with BMI and increased PDAC risk in human cohorts (Bao et al, 2013a). In mouse models of oncogenic *Kras*-driven PDAC, obesity (HFD and *Lep*^*ob/ob*^ induced) enhanced immune cell infiltration (predominantly myeloid cells including macrophages and neutrophils), pro-inflammatory cytokine expression (TNFα, IL-6, IL1β), and fibrosis (sirius red staining and αSMA+ myofibroblasts) in the microenvironment of emerging tumors (Khasawneh et al, 2009; Dawson et al, 2013; Philip et al, 2013; Incio et al, 2016a; Chang et al, 2017; Chung et al, 2020). Given the increasingly robust desmoplastic stroma observed as pancreatic tumors progress from low-grade to high-grade PanINs and ultimately to PDAC, it is challenging to disentangle whether the fibroinflammatory microenvironment is a cause or a consequence of enhanced tumor progression in obese models. In favor of the former, knockout of TNFα receptor (*TNFR1*) abrogated PanIN formation in HFD-fed *PK* mice (Khasawneh et al, 2009). Tumor formation in HFD-fed *EK-ER* mice was also impeded by inhibition of cyclooxygenase-2 (COX2) by conditional knockout (*Cox2*^*flox/flox*^) or pharmacologic blockade (using the non-steroidal anti-inflammatory drug celecoxib) (Philip et al, 2013). COX2 is a downstream target of KRAS and NFkB signaling in tumor cells and promotes the synthesis of proinflammatory prostaglandins that mediate local inflammation. A more recent study linking lineage-tracing to oncogene expression demonstrated that pretreatment with HFD feeding allows *Hras*^*G12V*^-transformed pancreatic duct epithelial cells to avoid apical expulsion, persist, and form tumors in *Ck19*^*CreERT*^*; Dnmt1*^*CAG-LSL-HrasG12V-IRES-GFP*^ (*CH*) mice. Anti-inflammatory treatment (in this case, with aspirin) reversed this phenotype and induced expulsion of transformed cells, providing a plausible mechanism by which HFD-induced local inflammation promotes tumorigenesis through retention of Ras-transformed cells (Sasaki et al, 2018). Contrary to this result, aspirin did not intercept tumor progression in *KCO* mice (Chung et al, 2020). Differences in the Ras mutant isoform (*Hras* versus *Kras*), specific cells in which oncogenic *Ras* was expressed (*Ck19*^*CreERT*^ in duct cells versus *Pdx1-Cre* in all pancreatic cells), oncogene expression levels (overexpression of *Hras* versus *Kras* expression from its endogenous locus), and timing of aspirin treatment may account for the divergent results. Nonetheless, these data argue that obesity elicits local inflammation to promote tumor initiation by Ras-transformed pancreatic epithelial cells (Fig 5A).

Obesity-induced reprogramming of the tumor microenvironment appears to have a similar pro-tumorigenic role later during tumor progression. By combining both autochthonous GEMMs and syngeneic transplant models, the Jain group demonstrated that obesity (HFD and *Lep*^*ob/ob*^ induced) enhances PDAC-associated desmoplasia via increased PSC activation by tumor-associated neutrophils (TANs) recruited by adipose-derived IL1β (Incio et al, 2016a). Suppression of PSC activation via angiotensin-receptor blockade, TAN depletion, or IL1β inhibition reversed the effect. Consistent with these models, increased collagen-I/hyaluronan-dense desmoplasia was present in human PDAC specimens from patients with elevated BMI (>30 kg/m^2^), leading to reduced chemotherapy response and worse outcomes (Incio et al, 2016a). A more recent study has corroborated these findings by demonstrating that HFD feeding promotes visceral adipose-mediated recruitment of TANs, which form neutrophil extracellular traps (NETs). Treatment with DNAseI to degrade NETs intercepted HFD-driven tumor progression (Wang et al, 2023), arguing that obesity-induced NETosis is required for the pro-tumorigenic effects of obesity, mirroring work in obesity-driven breast cancer metastasis (McDowell et al, 2021). An important contribution for myeloid infiltration in obesity-associated tumor growth was also shown in orthotopic transplants of pre-malignant *KC* and malignant *KPC* organoids (Lupo et al, 2020). HFD feeding enhanced CD11b^+^Ly6G^+^ TAN infiltration at the expense of CD8^+^ T cells as tumors progressed (*KC* to *KPC*), establishing an immunosuppressive microenvironment in obesity (Lupo et al, 2020). These data are consistent with other transplant models using traditional advanced PDAC cell lines that demonstrated increased myeloid infiltration and immunosuppressive cytokine expression in HFD-fed mice (Turbitt et al, 2019; Wogsland et al, 2021). HFD feeding has also been shown to enhance metastatic potential in syngeneic transplant models by reprogramming the immune microenvironment and metastatic niche. For example, tumor growth and mesenteric metastases from orthotopic transplants were increased by HFD-induced placental growth factor (PIGF)/VEGFR-1 signaling that repolarized macrophages towards a pro-tumorigenic phenotype (M2) in the tumor microenvironment (Incio et al, 2016b). Furthermore, HFD feeding increased liver metastases in an intrasplenic injection model via CX3CL1/CX3CR1 signaling resulting in PDAC cell recruitment (Sun et al, 2022), mirroring the formation of a pro-metastatic liver niche induced by IL-6 in nonobese *KPC* mice (Lee et al, 2019). As human obesity is also associated with increased local and systemic IL-6 levels (Kern et al, 2001), these data argue that altered cytokine production more broadly may drive niche formation to support metastatic spread in obesity. Despite obesity representing a systemic metabolic state of energy excess, its complex impacts on tumor cells, immune cells, adipocytes, and PSCs and their ensuing reciprocal interactions in mediating tumor initiation, progression, and metastasis (Fig 5B) are surprisingly local (Olson et al, 2017), arguing for the need to target the inflammatory microenvironment as a means to subvert obesity-driven PDAC.

The immunomodulatory effects of obesity must ultimately suppress adaptive immunity for tumors to develop and progress. Antibodies targeting the PD-1/PD-L1 immune checkpoint axis enable T cell-mediated anti-tumor immunity, yet they have been largely ineffective in most PDAC patients (O’Reilly et al, 2019); even patients with mismatch repair-deficient PDAC tumors and presumably higher tumor mutational burden showed reduced efficacy compared with patients with mismatch repair-deficient colorectal and endometrial cancers (Le et al, 2017; Maio et al, 2022). Nonetheless, several studies have demonstrated the importance of T cell-mediated immunity in shaping PDAC development and outcomes. For example, primary tumors from long-term PDAC survivors exhibited increased T cell immune responses and increased neoantigen epitope quality (Balachandran et al, 2017). Recurrent tumors in such long-term survivors (LTS) exhibited fewer and less immunogenic T cell neoantigens (Łuksza et al, 2022), consistent with immunoediting. Recent therapeutic studies have further shown that administration of autologous T cells expressing a TCR specific for a *KRAS* mutant epitope elicits an anti-tumor immune response (Leidner et al, 2022), whereas mRNA vaccines targeting patient-specific neoantigens led to immune responses concordant with prevention of relapse in half of patients after surgery in early-stage disease (Rojas et al, 2023). These data support the importance of T cell-mediated anti-tumor immunity in PDAC progression. However, most PDAC GEMMs lack neoantigens necessary to study anti-tumor T cell responses (Evans et al, 2016; Freed-Pastor et al, 2021) and the effects of obesity in shaping tumor immunity. Studies in carcinogen-induced or neoantigen-expressing transplant models of other tumor types harboring potent T cell neoantigens have suggested that HFD-induced obesity suppresses both the number and function of CD8^+^ cytotoxic T cells (Wang et al, 2019; Dyck et al, 2022), due in part to differential fatty acid uptake and partitioning between tumor and T cells within the tumor microenvironment (Ringel et al, 2020). Surprisingly, antibodies targeting the PD-1/PD-L1 immune checkpoint axis reversed T cell exhaustion and led to paradoxical enhancement of anti-tumor immunity in both obese mouse models and humans (Wang et al, 2019; Dyck et al, 2022). Therefore, understanding the mechanisms that link obesity to T cell dysfunction may have therapeutic relevance in multiple cancer types including PDAC, emphasizing the need for more faithful GEMMs to study neoantigen-specific T cell responses in obesity.

#### Microbial dysbiosis

Our gastrointestinal tract is colonized by a diverse array of bacterial, viral, fungal, and protozoan organisms, which comprise the microbiome and make up ∼99% of our genetic mass (Qin et al, 2010). Not surprisingly, alterations to our microbiome resulting in dysbiosis—loss of homeostasis—have been implicated in the etiology of a growing list of diseases including obesity and cancer. Therefore, recent studies have aimed to identify and characterize causal relationships between microbial alterations in the host, obesity, and cancer.

A series of groundbreaking experiments by the Gordon group first identified obesity-associated microbial patterns in obese humans and mice (Ley et al, 2005; Turnbaugh et al, 2006; Turnbaugh et al, 2008). These studies showed that the obesity-associated microbiome was significantly less diverse than that of lean controls and exhibited alterations in the ratio of specific bacterial phyla (Firmicutes to Bacteroides), which could be reversed by weight loss with caloric restriction or a low-fat diet. Fecal–microbial transplantation (FMT) from obese (*Lep*^*ob/ob*^) donor mice increased body fat in recipient mice (Turnbaugh et al, 2006), arguing that metabolic features of obesity may be transmissible via the gut microbiome. Similarly, experiments in mice subject to cyclic HFD feeding led to persistence of some microbial communities after weight loss that enhanced post-dieting weight gain, which could be transmitted via FMT (Thaiss et al, 2016). Although these results have not been fully mirrored in humans, some of the metabolic consequences of obesity, such as insulin resistance, were partially reversed by FMT from lean donors into obese individuals (Vrieze et al, 2012). Collectively, these results position the gut microbiome as a key player in obesity development and have ignited interest in microbiome modulation for the treatment of obesity and obesity-related diseases, such as cancer.

A growing body of literature has also shown an association between alterations in the microbiome and PDAC development. A meta-analysis of eight studies spanning over 200,000 participants found that patients who suffer from periodontal disease had a 70% increased risk of developing PDAC (Maisonneuve et al, 2017). This finding was supported by a subsequent study analyzing the relationship between oral pathogens and PDAC incidence in two independent cohorts of 732 participants (361 PDAC and 371 matched controls). Two specific species, *Porphyromonas gingivalis* and *Aggregatibacter actinomycetemcomitans*, were associated with higher PDAC risk (Fan et al, 2018). The mechanisms by which the oral microbiome promotes PDAC development have yet to be determined. Recent studies have also implicated the tumor microbiome in PDAC pathogenesis with greater tumor microbial diversity correlating with longer patient survival (Riquelme et al, 2019). Tumors from LTS showed greater CD3^+^ and CD8^+^ T cell infiltration than short-term survivors (STS), which correlated with the most enriched bacterial genus in LTS, arguing that the tumor microbiome might play an immunomodulatory role. Consistent with this hypothesis, FMT from LTS patients into mice harboring implanted *KPC* tumors showed enhanced immune infiltration and reduced tumor growth in a CD8^+^ T cell-dependent manner compared with FMT from STS and healthy control donors (Riquelme et al, 2019). These human studies led to a greater interest in developing models to study the mechanisms by which the microbiome may drive PDAC development with a focus on the gut and tumor microbiomes. Ablation of the microbiome—using antibiotic-treated or germ-free *KPC* mice—decreased PDAC formation (Pushalkar et al, 2018). Conversely, FMT of the gut microbiome from PDAC-harboring *KPC* mice markedly enhanced tumor progression compared with stool from WT (non-tumor-bearing) mice when transplanted into recipient *PK* mice. Analyses of intra-tumoral cell populations revealed that the PDAC-associated microbiome promoted recruitment of immunosuppressive macrophages, which mediated its pro-tumorigenic effects, providing a basis for targeting microbial communities and their capacity for immunomodulation to combat PDAC progression.

Beyond their effects on cancer progression, commensal bacteria have also been shown to modulate responses to cancer-directed therapies (chemotherapy and immunotherapy) by perturbing innate and adaptive immunity (Iida et al, 2013; Viaud et al, 2013) or altering the metabolism of cancer drugs (Geller et al, 2017). For example, researchers identified more than a dozen bacterial strains—present in the human microbiome—capable of metabolizing gemcitabine, a mainstay chemotherapeutic in PDAC therapy, to its inactive form. Bacteria could be found in more than three-quarters of human PDAC biospecimens and >90% of these bacterial samples tested rendered cell lines resistant to gemcitabine. Conversely, a bacterial metabolite, 3-IAA, was found to increase chemotherapeutic efficacy in orthotopic PDAC models and PDAC patients (Tintelnot et al, 2023).

Despite the plethora of evidence showing independent associations between obesity and PDAC with alterations of the microbiome, studies linking the obesity-associated microbiome to PDAC development are scarce. HFD diet feeding of *PK* mice resulted in changes in the gut microbiome that correlated with enhanced tumorigenesis, including increased abundance of specific species (e.g., *Clostridium sensu stricto*) (Dong et al, 2019). The findings were largely correlative and did not establish whether the microbial alterations were necessary or sufficient for HFD-driven tumorigenesis. In contrast, evidence from preclinical models of other gastrointestinal cancers (e.g., colorectal cancer, hepatocellular carcinoma) has more definitively established how HFD-induced microbial dysbiosis can modulate the immune system or elicit the production of specific bacterial metabolites to drive cancer development (Yoshimoto et al, 2013; Schulz et al, 2014). Given the wide availability of faithful models of both obesity and PDAC, it has become increasingly feasible to study the causal relationships between obesity, specific bacterial communities, and PDAC and to elucidate relevant pathogenic mechanisms (e.g., immunomodulation, metabolites). The potential pay-off could be new probiotic or antibiotic interventions for cancer prevention.

### Conclusions and future directions

Preclinical cancer modeling in mice has led to important discoveries into the causal mechanisms by which obesity promotes cancer progression, many of which are challenging to explore in humans. Yet, many important questions remain regarding the contributions of obesity to early tumor development. Systematic studies are necessary to untangle the differential roles of feeding behavior (e.g., overeating, intermittent fasting, etc.) and diet (fat source, nonfat macronutrients, etc.) on tumor development and whether physical activity can reverse these effects. Furthermore, there remains a dearth of studies examining putative mechanisms in early tumorigenesis, including (1) obesity-driven alterations in pre-tumor cell metabolism; (2) the range of pathogenic obesity-associated endocrine hormones, how they are induced, and how they stimulate tumorigenesis; (3) the precise cellular and molecular mechanisms by which obesity-induced inflammation drives tumor initiation and modulates anti-tumor immunity; and (4) whether and how obesity-associated microbial dysbiosis contributes to PDAC pathogenesis. Fortunately, the capacity to address these questions is being enabled by emerging isocaloric diet panels (Hu et al, 2018) and further advances in GEMMs and organoid transplant modeling (Tuveson & Clevers, 2019; Tuveson, 2021), such as reversible obesity models (Chung et al, 2020), defined mouse exercise paradigms (Kurz et al, 2022; Pita-Grisanti et al, 2023 *Preprint*), lineage-tracing of tumor progression (Rhim et al, 2012; Muzumdar et al, 2016), inducible neoantigen induction to study antigen-specific T cell responses in vivo (Damo et al, 2020; Freed-Pastor et al, 2021), and more sophisticated techniques to manipulate and analyze microbial communities (Chen et al, 2019; Han et al, 2021). With unbiased methods for epigenomic, transcriptomic, proteomic, and metabolomic analyses that now exist and increased funding from major organizations (Grand Challenges, National Cancer Institute, etc.), the time is ripe to dramatically expand our understanding of the obesity-cancer link, knowledge which will prove essential in combatting the rising scourge of obesity-driven cancer.
